# Stiffening of Circumferential F-Actin Bands Correlates With Regenerative Failure and May Act as a Biomechanical Brake in the Mammalian Inner Ear

**DOI:** 10.3389/fncel.2022.859882

**Published:** 2022-05-04

**Authors:** Mark A. Rudolf, Anna Andreeva, Christina E. Kim, Anthony C.-J. DeNovio, Antoan N. Koshar, Wendy Baker, Alexander X. Cartagena-Rivera, Jeffrey T. Corwin

**Affiliations:** ^1^Department of Neuroscience, University of Virginia School of Medicine, Charlottesville, VA, United States; ^2^School of Sciences and Humanities, Nazarbayev University, Nur-Sultan, Kazakhstan; ^3^Section on Mechanobiology, National Institute of Biomedical Imaging and Bioengineering, National Institutes of Health, Bethesda, MD, United States; ^4^Department of Cell Biology, University of Virginia School of Medicine, Charlottesville, VA, United States

**Keywords:** hair cell, utricle, regeneration, F-actin (filamentous actin), epithelial mechanics, alpha actinin 4, atomic force microscopy – AFM, micropipette aspiration (MPA)

## Abstract

The loss of inner ear hair cells causes permanent hearing and balance deficits in humans and other mammals, but non-mammals recover after supporting cells (SCs) divide and replace hair cells. The proliferative capacity of mammalian SCs declines as exceptionally thick circumferential F-actin bands develop at their adherens junctions. We hypothesized that the reinforced junctions were limiting regenerative responses of mammalian SCs by impeding changes in cell shape and epithelial tension. Using micropipette aspiration and atomic force microscopy, we measured mechanical properties of utricles from mice and chickens. Our data show that the epithelial surface of the mouse utricle stiffens significantly during postnatal maturation. This stiffening correlates with and is dependent on the postnatal accumulation of F-actin and the cross-linker Alpha-Actinin-4 at SC-SC junctions. In chicken utricles, where SCs lack junctional reinforcement, the epithelial surface remains compliant. There, SCs undergo oriented cell divisions and their apical surfaces progressively elongate throughout development, consistent with anisotropic intraepithelial tension. In chicken utricles, inhibition of actomyosin contractility led to drastic SC shape change and epithelial buckling, but neither occurred in mouse utricles. These findings suggest that species differences in the capacity for hair cell regeneration may be attributable in part to the differences in the stiffness and contractility of the actin cytoskeletal elements that reinforce adherens junctions and participate in regulation of the cell cycle.

## Introduction

Permanent hearing loss affects more than 400 million people worldwide, and studies indicate that vestibular dysfunctions affect roughly one third of adults (Agrawal et al., 2009; World Health Organization [WHO], 2018). The loss of sensory hair cells (HCs) in the inner ear is a major irreversible cause of these sensory deficits. HCs are vulnerable to loud sound, ototoxic drugs, and aging, and are not effectively replaced in humans and other mammals (Nadol, 1993). In contrast, HC loss in fish, amphibians, and birds causes neighboring supporting cells (SCs) to divide and produce progeny that can differentiate into replacement HCs, and in some cases convert directly into HCs (Corwin and Cotanche, 1988; Baird et al., 1996). What accounts for the difference in regenerative capacity has remained unclear.

Studies of epithelial repair have shown that mechanical forces arising from cell loss propagate through cell-cell junctions, increasing tension in the cortical cytoskeletons and changing the shapes of neighboring cells, which activates mechanosensitive signaling molecules such as YAP/TAZ and leads to cell proliferation (Rauskolb et al., 2014; Benham-Pyle et al., 2015). Past research has shown that the intercellular junctions of SCs in the chicken utricle contain little or no E-cadherin and are associated with thin circumferential “belts” of F-actin as are epithelial cells in general. In contrast, utricular SCs in mice and humans develop E-cadherin-rich junctions that are bracketed by exceptionally wide and thick regions of circumferential F-actin (Burns et al., 2008; Burns and Corwin, 2013). Throughout this article we use the term “F-actin bands” as a more appropriate descriptor for the exceptionally robust structures in mouse SCs. The growth of the circumferential F-actin bands in SCs of the mouse utricle strongly correlates to declines in the capacity for the cells to change shape (*R* = −0.989) and proliferate (*R* = −0.975) in culture (Burns et al., 2008; Collado et al., 2011b). HC loss and SC shape change also have been found to activate YAP in SCs of the chicken utricle, but lead to little or no nuclear accumulation in the mouse utricle (Rudolf et al., 2020; Borse et al., 2021). Those findings suggested that the thin F-actin belts in non-mammalian SCs might more readily permit shape changes and proliferation, whereas the thick F-actin bands in mammalian SCs could restrict or slow deformations, thereby reducing signals that can evoke proliferative responses.

To test our hypothesis that the thick F-actin bands limit deformation of mammalian SCs, we measured stiffness of the epithelial surface in utricles from mice and chickens of various ages using atomic force microscopy (AFM) and micropipette aspiration. The measurements revealed that the mouse utricular epithelium stiffens postnatally, particularly at SC junctions. F-actin and the cross-linker Alpha-Actinin-4 substantially contribute to the measured stiffness. In stark contrast, chicken utricles remained relatively compliant. Analysis of cell shape revealed that the apical domains of chicken SCs elongate and align throughout development. Inhibition of myosin II contractility led to dramatic expansion of chicken SC surfaces, but it did not affect the size and regular shapes of mouse SCs. The results indicate that the exceptionally thick and cross-linked circumferential F-actin bands stiffen mammalian SCs and appear likely to limit the mechanical signals produced during cell loss, whereas the compliant SCs in birds exhibit collective mechanical behavior that is favorable for epithelial repair.

## Materials and Methods

### Animals and Dissection of Vestibular Organs

All animals were handled in accordance with protocols approved by the Animal Care and Use Committee at the University of Virginia (protocol 18350718) and NIH guidelines for animal use (protocol 1254-18). Utricles from embryonic day 16 (E16), postnatal day zero (P0), or adult (>6 week old) mice were used with staging of embryos according to The Atlas of Mouse Development (Kaufman, 1992). Swiss Webster mice were obtained from Charles River Laboratories. Transgenic mice that express a γ-actin and green fluorescent protein fusion (γ-actin–GFP) were generated in the laboratory of Dr. Andrew Matus (Fischer et al., 1998). Mice with whole-body knockout of *Alpha-Actinin-4 (Actn4* KO) were generated in the laboratory of Dr. Martin Pollak (Kos et al., 2003). Fertilized White Leghorn (W-36) eggs were obtained from Hy-Line and incubated at 37°C in a humidified chamber with rocking until E18, after which eggs were incubated without rocking. Embryos were staged according to morphometric features (Hamburger and Hamilton, 1951). Animals of either sex were used for all experiments.

For tissue harvest, labyrinths were dissected from temporal bones in ice-cold, HEPES-buffered DMEM/F-12 (Thermo Fisher Scientific), utricles were isolated, and the roof and the otoconia were mechanically removed.

### Generation of *Actn4* Conditional Knockout Mice

To generate *Actn4^flox/flox^* mice, frozen sperm from *Actn4^TM 1a(EUCOMM)Wtsi^* mice (Mouse Genome Informatics ID: 4441842) were obtained from the European Mouse Mutant Archive (EMMA) (EM:05964). The *Actn4^TM 1a(EUCOMM)Wtsi^* mice harbor a loxP site downstream of *Actn4* exon 5 at position 28615171 of chromosome 7 (Build GRCm39), as well as an L1L2_gt1 cassette upstream of exon 5 at position 28614391, which contains an FRT-flanked lacZ/neomycin sequence followed by a loxP site. The Genetically Engineered Murine Model core at the University of Virginia performed *in vitro* fertilization to rederive the line, as previously described (Takeo and Nakagata, 2011). The conditional knockout line was then generated per the EMMA protocol. Briefly, FLP germline deleter mice were crossed with the *Actn4^TM 1a(EUCOMM)Wtsi^* mice to excise the FRT-flanked lacZ/neomycin sequence, producing mice with an allele of *Actn4* in which the critical exon 5 is floxed. The resulting *Actn4^flox/flox^* mice were crossed to 129(Cg)-*Foxg1^TM 1(cre)Skm^*/J mice (*Foxg1-Cre*, Jackson Laboratory stock 004337) which express Cre recombinase in the embryonic forebrain and otic vesicle (Hébert and McConnell, 2000), producing *Actn4*^flox/flox^*;Foxg1-Cre* (*Actn4* cKO) mice. *Actn4^flox/flox^* mice were genotyped using the following primers: 5′-AACTCAGGATGGAGTTGGGC-3′ (common forward), 5′-TGGATGTGGGTGATCTTTGC-3′ (wild type reverse), and 5′-TCGTGGTATCGTTATGCGCC-3′ (floxed reverse). An amplicon measuring 455 bp signifies a wild type allele of *Actn4*, and an amplicon of 184 bp signifies a floxed allele. *Foxg1-Cre* mice were genotyped using the following primers: 5′-AGAACCTGAAGATGTTCGCG-3′ (*Cre* forward) and 5′-GGCTATACGTAACAGGGTGT-3′ (*Cre* reverse). An amplicon measuring 328 bp signifies the presence of a *Cre* allele.

### Organ Culture

Utricles dissected from mice and chickens under aseptic conditions were transferred to glass-bottom dishes (Mat-Tek) or Willco dishes (Willco Wells) coated with Cell-Tak (1 μL air-dried onto the glass; BD Biosciences #354240) and adhered with the stromal side down. Utricles were cultured in DMEM/F-12 + HEPES with 1% fetal bovine serum (FBS; Invitrogen) and 10 μg/ml Ciprofloxacin (Bayer) at 37°C and 5% CO_2_. In some experiments, cytochalasin D (Sigma-Aldrich), blebbistatin (Calbiochem), or streptomycin sulfate (Sigma) were added to the culture medium at the concentrations and durations indicated below.

### Micropipette Aspiration

To measure the mechanical stability of sensory epithelia from chickens and mice of various ages, we adapted micropipette aspiration to quantify tissue-level resistance to deformation in utricles. This technique enables direct observation of deformation lengths produced by a calibrated suction, and can be applied in length scales ranging from single cells (Hochmuth, 2000) to whole embryos (Porazinski et al., 2015). Two types of micropipettes were fabricated from borosilicate glass tubes on a Sutter Instruments puller: “stabilizing pipettes” were scored with a tile and broken to a clean edge of 50–100 μm internal diameter, and “aspiration pipettes” were scored with a tile and broken to a clean edge of 22.5 μm internal diameter (which spans ∼5 HCs). The tip of the aspiration micropipette was dipped in Sigmacote (Sigma SL2) and allowed to dry before use in order to minimize adhesion to debris.

**FIGURE 1:**
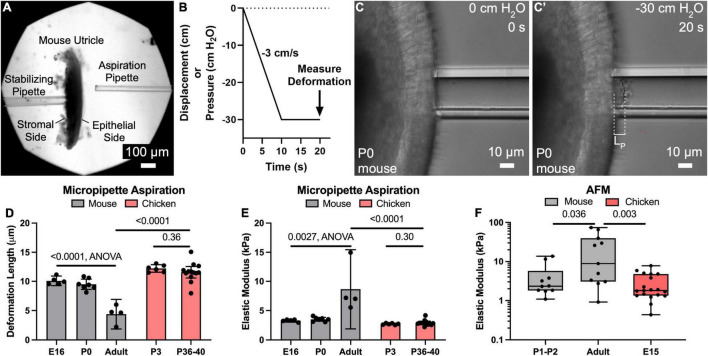
Micropipette aspiration and AFM measurements reveal that the apical surface of utricles from mature mice are significantly more resistant to deformation than those of newborn mice and chickens. **(A)** Low magnification image of a mouse utricle prior to micropipette aspiration. A stabilizing pipette ∼50–100 μm in diameter (left) was used to apply light suction on the “stromal side” to maintain the utricle in place. Another pipette 22.5 μm (∼5 cell apices) in diameter (right) was used to apply a defined negative pressure to the epithelial surface of the utricle. **(B)** The negative pressure was ramped from 0 cm H_2_O to –30 cm H_2_O over a span of 10 s, and then maintained at –30 cm H_2_O for an additional 10 s. The deformation length of the apical surface was after the full 20 s “pull” was recorded. **(C,C’)** High magnification images of the apical surface of a P0 mouse utricle during application of negative pressure, producing a deformation of length L_P_. **(D)** Plot of mean deformation length of utricles from mice and chickens of various ages. Error bars denote 95% confidence intervals. Dots represent an average of at least three measurements from a single utricle. *P*-values are listed from unpaired *t*-tests or ANOVA. **(E)** Plot of elastic moduli as measured by micropipette aspiration of the utricular epithelial surface, as calculated from the deformation lengths in **(D)**. Error bars denote 95% confidence intervals, and each dot represents one utricle. *P*-values are listed from unpaired *t*-tests or ANOVA. **(F)** Boxplot in logarithmic scale of elastic moduli as measured by AFM of the utricular epithelial surface. Each dot represents the average elastic modulus obtained from an eight-point line scan. *P*-values are indicated from Mann-Whitney tests.

On the day of tissue harvest, utricles for micropipette aspiration measurement were cultured atop 0.08 μm Nucleopore filters (Whatman) at the meniscus of the culture medium. On the following day, utricles were transferred to a glass-bottom POCR chamber (Zeiss) containing DMEM/F12 + HEPES supplemented with 5% FBS, and the utricle was adhered to the Cell-Tak-coated glass with the stromal side down. Under an upright microscope with a heated stage set to 37°C, the utricle was visualized with a 2× objective and partially lifted from the glass using fine forceps, with one edge remaining adhered to the glass. The “stabilizing” pipette of 50–100 μm internal diameter was positioned via a micromanipulator into contact with the stromal side of the utricle and held the macular surface parallel to the optical axis (Figure 1A). The second, “aspiration” micropipette of 22.5 μm internal diameter was used for micropipette aspiration measurements of the apical surface. Water-filled tubing connected each micropipette to an open-barreled syringe (water reservoir) that was raised or lowered to control suction and serve as a manometer. A 60×, 0.9 NA water immersion objective and differential interference contrast were used to visualize the utricle’s apical surface and the tip of the micropipette.

To perform a measurement, the meniscus of the water reservoir was initially held at the same height as the utricle, so that no suction pressure would be present upon contact. Then, a micromanipulator was used to bring the aspiration pipette into light contact with the sensory epithelium’s apical surface. The water reservoir was then lowered to apply a ramped negative pressure, resulting in deformation of the apical surface that could be visualized with differential interference contrast video microscopy (Figures 1B,C; Supplementary Movie 1). Specifically, the water reservoir was lowered at a rate of 3 cm/s for 10 s (equivalent to a negative pressure ramp of 3 cm H_2_O/s or ∼300 Pa/s), and then held with the meniscus 30 cm below the height of the utricle (equivalent to a negative pressure of 30 cm H_2_O or ∼3 kPa) for an additional 10 s. As the utricle’s apical surface deformed during that process, images of the deformation were acquired at 1 s increments using a SPOT Idea CMOS camera. After the 20 s acquisition, the final deformation length was measured in a blinded fashion using FIJI software (Schindelin et al., 2012). Each utricle was measured at least three times, and the individual measurements from each utricle were averaged to generate a single value of deformation length, which was considered a single independent observation for statistical analysis.

Elastic Young’s modulus (*E*; Pa) was calculated using a simple continuum model that treats the tissue as a homogeneous, incompressible, linear-elastic half space (Theret et al., 1988; González-Bermúdez et al., 2019):


$$E_{\mathrm{tissue}}=\Delta \,P\,\frac{3\,\varphi_{p}}{2\,\pi }\,\frac{R_{p}}{L_{p}}$$

In this equation, the applied pressure gradient Δ*P* was 30 cm H_2_O (2942 Pa), the pipette wall thickness parameter *φ_*p*_* was set to 2.1, the pipette inner radius *R*_*p*_ was 11.25 μm, and *L*_*p*_ was the measured deformation length (μm). The average deformation length was used to calculate one value of *E* per utricle, which was considered a single independent observation for statistical analysis.

### Atomic Force Microscopy

Atomic force microscopy (AFM) measurements were conducted for utricular sensory epithelia of CD-1 mice (Charles River Laboratories) and embryonic Delaware chickens (B & E eggs, PA). To comply with the language of the animal protocol at the NIH, utricles were harvested exclusively from embryonic and not post-hatch chickens. Eggs were incubated at 37.5°C in an automatic rocking incubator (Brinsea), and all embryos were terminated prior to hatching at E21. Utricles were freshly dissected as above and mounted on glass-bottom dishes (HBST-5040, Willco Wells) precoated with 2 μl of Cell-Tak (Corning) and immersed in Leibovitz media (L-15, 21083-027, Life Technologies). AFM measurements were obtained on a Bioscope Catalyst AFM system (Bruker) mounted on an inverted Axiovert 200M microscope (Zeiss) equipped with a confocal laser scanning microscope (510 Meta, Zeiss) and a 40× objective lens (0.95 NA, Plan-Apochromat, Zeiss). Explants were maintained at 37°C using a Bruker heated stage. For tissue-level measurements, line scans of eight points spaced between 500 nm and 1 μm apart were obtained from the apical surface of the sensory epithelium. We used soft silicon nitride Bruker MLCT AFM probes with nominal tip radius of 20 nm. The MLCT cantilevers were precalibrated by using the AFM built-in thermal method, yielding spring constants between 0.07 and 0.12 N/m. For some experiments, sheets of utricular sensory epithelium were obtained by incubating the utricles in 0.5% thermolysin at 37°C for 15 min, and mechanically separating the epithelium from the underlying stroma. PeakForce Tapping (PFT) Quantitative Imaging mode was used for high-resolution measurements, as has been performed previously for living inner ear tissues (Theret et al., 1988; Cartagena-Rivera et al., 2019; González-Bermúdez et al., 2019; Katsuno et al., 2019). The AFM probe specially designed for live cell imaging (PFQNM-LC, Bruker) has a tip height of 17 μm, controlled tip radius of 65 nm, and opening angle of 15°. The cantilevers (Bruker, PFQNM-LC-A-CAL) were precalibrated right after manufacturing by Bruker using a laser Doppler vibrometer. We confirmed the precalibration by Bruker using the AFM built-in thermal tuning method, and the obtained spring constant range of the cantilevers was between 0.06 and 0.08 N/m. For PFT-AFM imaging, we used a driving frequency of 500 Hz and drive amplitudes of 500 to 650 nm. The scan speed used was 0.5 Hz. The PeakForce feedback was set between 800 pN and 1.2 nN. The elastic Young’s modulus (stiffness) calculations for both AFM modalities used in this study were performed using NanoScope Analysis software (Bruker). The elastic Young’s modulus (*E*) was computed by fitting each force-distance curve with the Sneddon’s contact mechanics model for indenting an infinite isotropic elastic half-space with a conical indenter:


$$F_{\mathrm{Sneddon}}=\left(8\,E\,\tan\,\frac{\alpha }{3}\,\pi \right)\,\delta^{2}$$

In this equation, *F* is the applied force, α is the tip half-opening angle, and δ is the sample mean indentation. A single value of *E* was obtained for each line scan by averaging the eight values that comprised each line scan. One line scan was considered an independent replicate for statistical analysis.

### Immunohistochemistry

Utricles were fixed in 4% paraformaldehyde for 1 h at room temperature or Shandon Glyo-Fixx overnight at 4°C. After washing in PBS with 0.1% Triton X-100 (PBST), utricles were blocked in 10% normal goat serum (Vector) in PBST for 2 h at room temperature. Primary antibodies were incubated with 2% normal goat serum at 4°C overnight. After washing in PBST, secondary antibodies were added in 2% normal goat serum and incubated for 2 h at room temperature. Where indicated below, AlexaFluor-conjugated phalloidin (1:100) and Hoechst 33452 (1:500) were incubated along with secondary antibodies to detect actin and nuclei. After washing in PBST, samples were mounted in Prolong Diamond and imaged using a Zeiss 880 confocal microscope or a Zeiss Axiovert 200M widefield microscope. Primary antibodies used included: Rabbit anti-Calretinin (Millipore Sigma AB5054, 1:200), mouse anti-N-cadherin (BD Biosciences 610920, 1:200), rabbit anti-ZO-1 (Thermo Fisher Scientific 40-2200, 1:200), rabbit anti-Chick Occludin (generous gift from Shoichiro Tsukita, 1:200), mouse anti-Spectrin (Millipore MAB1622, 1:200), rabbit anti-phospho-Histone H3 (Ser10) (Millipore 06-570, 1:400), rabbit anti-Cingulin (Zymed 36-4401, 1:200), and rabbit anti-Filamin A (Abcam ab51217, 1:200).

### RNA Extraction and Quantitative PCR

To extract RNA, 10 mouse utricles or 2–4 chicken utricles were harvested, pooled, and dissolved in 500 μL TRI-reagent (Molecular Research Center). RNA was precipitated using 2 μL of polyacryl carrier (Molecular Research Center) per the manufacturer’s protocol, and cDNA was generated with a High-Capacity RNA-to-cDNA kit (Applied Biosystems). Quantitative PCR was performed using SensiMix SYBR Green and Fluorescein kit (Quantace) on a MyIQ/iCycler (Bio-Rad). Two technical replicates were used for each run. The Miner algorithm was used to analyze gene expression (Zhao and Fernald, 2005). For mouse utricles, *Ppia* (also known as *Cyclophilin A*) served as an endogenous reference gene (forward primer, CAGTGCTCAGAGCTCGAAAGT; reverse primer, GTGTTCTTCGACATCACGGC), and *Actn4* was the target gene (forward primer, TCCACTTACAGACATCGTGAACACA; reverse primer, GCATGGTAGAAGCTGGACACATAT). For chicken utricles, *ACTB* served as an endogenous reference gene (forward primer, CCGGCTCTGACTGACCGCGT; reverse primer, GGCATCGTCCCCGGCGAAAC), and *ACTN4* was the target gene (forward primer, ACGGAGCGCCATGGTGGATT; reverse primer, AGCAGCAGGTCTCGGTCCCA). Three biological replicates were acquired for mouse samples, and two biological replicates were acquired for chicken samples. An unpaired *t*-test was used for statistical comparison of gene expression between different ages.

### Measurement of Macular Area

After performing micropipette aspiration measurements, utricles of adult control and *Actn4 cKO* mice were labeled with phalloidin and imaged on a Zeiss 880 confocal microscope. Maximum intensity projections were generated in FIJI, and outlines of the utricular maculae were hand-drawn by using stereociliary bundles to determine the macular border. The macular areas of the left and right utricles were averaged together to produce one value of macular area per mouse, which was considered an independent biological replicate for statistical analysis.

### Transmission Electron Microscopy

Transmission electron microscopy (TEM) images of the apical junctional region of utricles from *Actn4* KO mice were obtained as previously described (Burns et al., 2008). Briefly, mice were euthanized as above, and within 3 min a fixative solution composed of 3% glutaraldehyde in 0.15 M sodium cacodylate buffer at pH 7.4 was injected into the superior semicircular canal to replace the endolymph. The labyrinth was then isolated and fixed overnight at 4°C. Then the utricle was dissected in 0.15 M cacodylate buffer and post-fixed in 1% osmium-tetroxide. The tissue was dehydrated in a graded ethanol series and infiltrated with Epon 812 (Electron Microscopy Sciences, Hatfield, PA, United States). Thin sections were cut in the plane parallel to the epithelial surface with a diamond knife. Sections were collected on copper grids and stained 5 min with lead citrate, 15 min with uranyl acetate, and 5 min with lead citrate. A JEOL 1230 transmission electron microscope was used for imaging.

### Cell Shape Measurements

Supporting cell apical junctions were visualized by immunostaining for ZO-1 or N-cadherin in mouse utricles and immunostaining for cingulin, occludin, or N-cadherin in chicken utricles. For cell shape measurements, five images were acquired per utricle with 30 contiguous SCs quantified from the center of each image, all of which were pooled and considered an independent observation. Mouse utricles were imaged in five standardized locations (lateral extrastriolar, anterior, medial, posterior, and central (striolar) regions). All images of the chicken utricle were acquired in the large, medial extrastriolar region.

All image analysis was done using FIJI. First, SC apical domains were hand-traced from high-magnification images of the utricular apical surface, with 30 contiguous cells analyzed from the center of each image. The apical domain area of each SC was measured. Then, each SC apical domain was fit to an ellipse using the software’s built-in function, which provided the length of each apical domain’s major axis, minor axis, and angular orientation. Elongation (length:width ratio) was calculated from the ratio of the major and minor axes of each apical domain. The intercellular alignment for a given image was calculated using the following equation, where *n* is the number of apical domains analyzed in the image (30), θ*_*med*_* is the median ellipse orientation for a given image, and θ*_*i*_* is the orientation of ellipse *i*.


$$\mathrm{Intercellular}\,\mathrm{alignment}=\frac{1}{n}\,\sum_{i=1}^{n}\left|\theta_{\mathrm{med}}-\theta_{i}\right|$$

Using this definition, perfectly aligned cells have a value of 0° deviation from the median angle, and randomly aligned cells have an intercellular alignment approaching 45° deviation from the median angle.

### Measurement of Cell Division Orientation

To measure cell division orientation in the developing chicken utricle, utricles were harvested at E14 and immunostained for phospho-histone H3 (PH3) to label dividing cells and spectrin to label the cuticular plates of HCs. Using an Axiovert 200M inverted microscope, cells in metaphase and anaphase were identified. Each mitotic figure was imaged together with nearby HC cuticular plates, which were used to determine the local axis of HC polarity. The division axis of a cell in metaphase was determined by drawing a line that spanned the metaphase plate, measuring its angle in FIJI, and adding 90° to account for the fact that metaphase cells divide perpendicular to the orientation of their metaphase plate. The division axis of a cell in anaphase was determined by drawing a line from one pole to the other and measuring its angle in FIJI. The local axis of HC polarity was determined by averaging the polarity of the six HCs closest to each mitotic figure. Each HC’s polarity was measured by drawing a line that bisected its spectrin-labeled cuticular plate and terminated at the fonticulus, and the resulting angle was measured in FIJI. The difference between each cell’s division axis and the axis of local HC polarity was recorded as “degrees deviation from local HC polarity.” Using this definition, the cell division orientation can range from 0° (division axis parallel to the local axis of HC polarity) to 90° (division axis perpendicular to the local axis of HC polarity).

To measure cell division orientation in the regenerating chicken utricle, utricles from P2 chickens were harvested and cultured 24 h in medium containing 1 mM streptomycin sulfate to ablate HCs. Utricles were then transferred to a dish containing standard culture medium and incubated an additional 48 h prior to fixation. Anti-cingulin was used to label cell junctions, and Hoechst 33342 was used to label nuclei. The orientations of dividing cells in metaphase or anaphase were measured as described above. Because HCs were ablated, the orientation of cell divisions was measured with respect to the long axis of neighboring SCs, which aligns with the axis of HC polarity. To do this, the apical domains of the six closest SCs were outlined using cingulin immunoreactivity and fit to an ellipse using FIJI. Then, the orientations of the major axes of the ellipses were averaged together. The difference between a given cell’s division axis and the local axis of SC elongation was recorded as “degrees deviation from local SC elongation.” Using this definition, the cell division orientation can range from 0° (division axis parallel to the local axis of SC elongation) to 90° (division axis perpendicular to the local axis of SC elongation).

### Time-Lapse Imaging

Chicken utricles were harvested and incubated in culture medium containing 100 nM SiR-actin (Cytoskeleton CY-SC006) for 6 h to label F-actin. They were then transferred to DMEM/F-12 + HEPES containing 3 μM FM1-43FX (Thermo Fisher F35355) for 3 min to label HCs. For imaging, utricles were transferred to a Zeiss POCR chamber with a 42 mm coverglass that contained DMEM/F-12 + HEPES supplemented with 10% FBS. A wax barrier bisected the chamber for the simultaneous imaging of two utricles. Utricles were immobilized HC-side down with a miniature “harp” (gold wire threaded with fine nylon) as previously described (Bird et al., 2010). Then, the imaging chamber was placed on a 37°C heated stage insert supplemented with 5% CO_2_. Just prior to image acquisition, blebbistatin was spiked into one side of the bisected chamber at a final concentration of 50 μM, and DMSO was added to the second side as a vehicle control. The time-lapse was acquired on a Zeiss 880 confocal microscope, with z-stacks obtained at 10 min intervals for a duration of 9 h.

### Statistics

GraphPad Prism 9 software was used for statistical tests. Unpaired student’s *t*-tests or Mann-Whitney tests were used for pairwise comparisons. ANOVA was used to analyze comparisons among three or more groups, and Tukey’s test was applied where indicated. The Kolmogorov-Smirnov (KS) test was used to test whether cell division orientations were randomly oriented, with a uniform distribution as the null hypothesis. Unless otherwise stated, bar graphs display mean ± 95% confidence interval, with dots denoting independent biological replicates. Test statistics, sample sizes, degrees of freedom, and *p*-values are reported in the Results, on graphs, or in the figure legends. For all tests, a *p*-value less than 0.05 was considered statistically significant.

## Results

### Utricular Sensory Epithelia of Mature Mice Are More Resistant to Deformation Than Those of Developing Mice and Chickens

To test the hypothesis that the surface of the sensory epithelium would become more resistant to deformation as mice develop and mature, we used the customized micropipette aspiration described above in utricles from E16, P0, or adults > 6 weeks old (Figures 1A–C). Similar deformation lengths were measured in E16 and P0 mouse utricles (10.1 ± 0.8 μm and 9.5 ± 0.9 μm, mean ± 95% confidence interval), corresponding to tissue-level elastic moduli of 3.3 ± 0.3 kPa and 3.5 ± 0.3 kPa, respectively. In contrast, utricles from adults deformed less than half as much (4.4 ± 2.5 μm; *p* < 0.0001, *F*_(2,13)_ = 38.16, ANOVA, *n* = 4–7 utricles per condition, Figure 1D), which corresponds to a 2.5-fold increase in elastic modulus compared to that measured in utricles from newborn mice (8.7 ± 6.8 kPa; *p* = 0.0027, *F*_(2,13)_ = 2.24, ANOVA, *n* = 4–7 utricles per condition, Figure 1E).

To compare the stiffness of mouse utricles to measurements from a non-mammalian vertebrate that readily regenerates hair cells, we applied the same technique in utricles from P3 and P36-40 chickens. Despite the age difference, the measurements of mean deformation lengths showed no significant difference (12.2 ± 0.7 μm vs. 11.6 ± 1.0 μm, *p* = 0.36, *t*_(17)_ = 0.93, unpaired *t*-test, *n* = 6–13 utricles per condition, Figure 1D), and no significant difference in calculated elastic moduli (2.7 ± 0.1 kPa vs. 2.9 ± 0.3 kPa, *p* = 0.31, *t*_(17)_ = 1.06, unpaired *t*-test, *n* = 6–13 utricles per condition, Figure 1E). Moreover, the deformation lengths of P36-40 chicken utricles were 2.6-fold greater than those measured for adult mice (*p* < 0.0001, *t*_(15)_ = 7.7, unpaired *t*-test, *n* = 4–13 utricles per condition, Figure 1D), which corresponds to an elastic modulus ∼1/3 of that for their mammalian counterparts (*p* < 0.0001, *t*_(15)_ = 7.2, unpaired *t*-test, *n* = 4–13 utricles per condition, Figure 1E).

To independently assess surface mechanical properties of utricles from neonatal mice, adult mice, and embryonic chickens, we used AFM. Consistent with the results from micropipette aspiration, AFM yielded a median elastic modulus for the utricles from adult mice that was 3.8-fold greater than that for P1-2 mice (8.8 kPa vs. 2.3 kPa, *p* = 0.036, Mann-Whitney test, *n* = 10–11 line scans per condition, Figure 1F) and 4.9-fold greater than that for utricles from E15 chickens (8.8 kPa vs. 1.8 kPa, *p* = 0.0033, Mann-Whitney test, *n* = 11–19 line scans per condition, Figure 1F). When we used AFM to measure the surface of delaminated sheets of utricular epithelium, values obtained were not significantly different than the AFM measurements from the surfaces of utricles which contained the underlying stroma (mouse: *p* = 0.43, chicken: *p* = 0.46, Mann-Whitney test, *n* = 6–19 line scans per condition, Supplementary Figure 1). This indicates that the original measurements made in utricles containing the stroma reflect the mechanical properties of the sensory epithelium itself. Furthermore, calculated elastic moduli from micropipette aspiration measurements were not significantly different than those obtained from AFM (P0-P2 mice: 3.5 kPa vs. 2.3 kPa median elastic modulus, *p* = 0.23, *p* = 0.95, Mann-Whitney test, *n* = 4–11 measurements). Taken together, the data indicate that maturational changes in the mouse utricle result in the sensory epithelium developing greater resistance to deformation than the sensory epithelium of the chicken utricle, where we did not detect postembryonic stiffening.

### F-Actin Depolymerization and Deletion of *Actn4* Reduce the Stiffness of Mature Mouse Utricles

**FIGURE 2:**
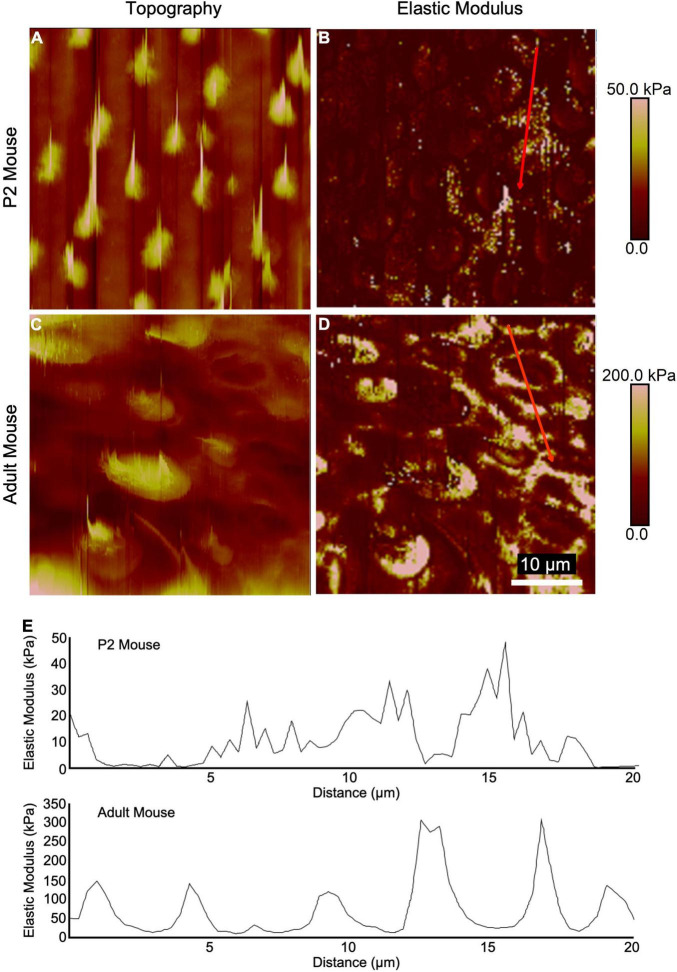
High-resolution AFM measurements of utricular sensory epithelia from neonatal and adult mice show elevated stiffness at intercellular junctions. **(A,C)** Topography mapping of the utricular surface from P2 and adult mice reveals locations of hair bundles. **(B,D)** Elastic modulus mapping of the utricular surface shows elevated stiffnesses in regions corresponding to HC cuticular plates and intercellular junctions. **(E)** Line scans depicting elastic modulus along the red traces in **(B,D)**.

High-resolution AFM measurements and topography mapping at the epithelial surface of utricles from neonatal and adult mice revealed apical protrusions corresponding to HC stereociliary bundles (Figures 2A,C). In adult mouse utricles, the measured elastic moduli at the intercellular junctions of SCs often exceeded 100 kPa with maximum peaks reaching ∼300 kPa (Figures 2D,E), while corresponding regions in P2 mouse utricles rarely exceeded 50 kPa (Figures 2B,E). The results indicate that the intercellular junctions in mouse vestibular SCs become significantly stiffer with age, and thus more resistant to changes in cell shape.

**FIGURE 3:**
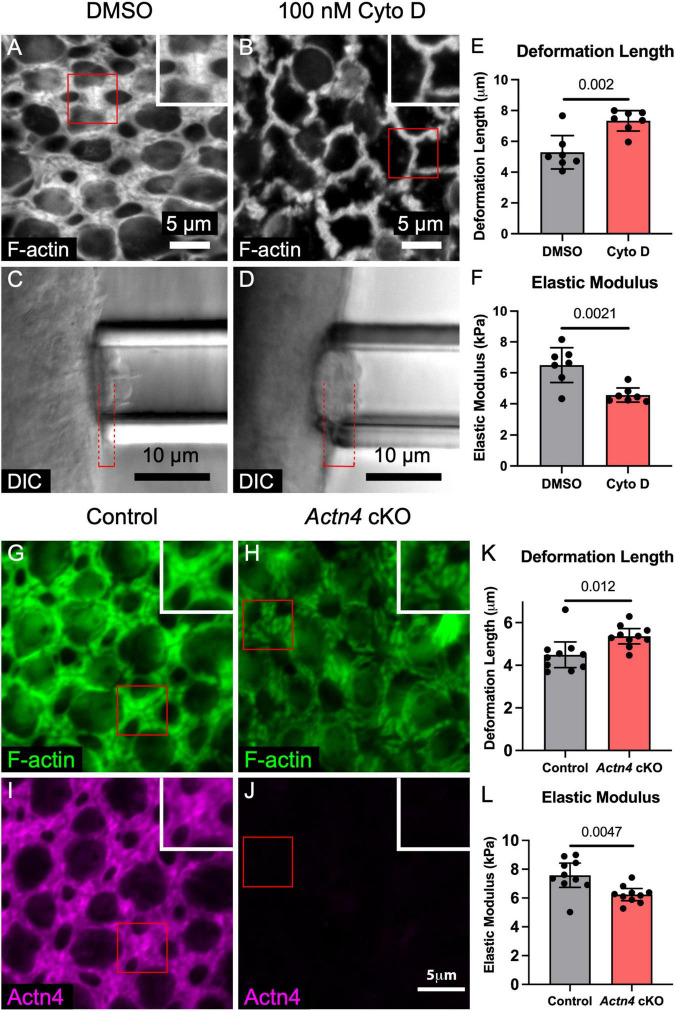
F-actin depolymerization and deletion of the cross-linker *Actn4* each disrupt the morphology of the circumferential F-actin bands in SCs of adult mouse utricles and reduce stiffness of the apical surface. **(A,B)** Representative images of phalloidin-labeled F-actin in utricles from adult mice that were cultured for 24 h in DMSO or 100 nM cytochalasin D (Cyto D), an F-actin depolymerizing agent. Insets depict higher-magnification views of a SC-SC junction. **(C,D)** Micropipette aspiration of utricles from adult mice that were cultured in DMSO or 100 nM Cyto D for 24 h. Red dashed lines denote the deformation lengths. **(E)** Blinded quantification revealed that the mean deformation length of utricles cultured in Cyto D was significantly greater than that of DMSO-treated controls. Dots represent an average of at least three measurements from a single utricle. **(F)** Quantification of elastic modulus revealed a significant difference in stiffness. **(G,H)** Circumferential F-actin bands were normal in utricles of control *Actn4^flox/flox^* mice, but those in *Actn4* cKO mice had disrupted morphology. **(I,J)** Actn4 immunostaining labeled the F-actin bands in control mice, but the F-actin bands in Actn4 cKO mice had sharply reduced immunoreactivity. **(K,L)** Quantification of deformation length and elastic modulus from micropipette aspiration of utricles from *Actn4* cKO mice and littermate controls. All data are shown as mean ± 95% confidence interval.

Imaging during micropipette aspiration measurements in utricles from P0 and adult γ-actin–GFP mice showed that F-actin at the apices of SCs is deformed, consistent with a hypothesized contribution to stiffness (Supplementary Movie 2). To test whether the stiffness of the mouse utricular epithelium is dependent on F-actin, we cultured utricles from adult mice in the presence of 100 nM cytochalasin D for 24 h, which depolymerized the F-actin bands in SCs (Figures 3A,B; Burns and Corwin, 2014). Micropipette aspiration revealed that cytochalasin D treatment resulted in a nearly 40% increase in deformation length over DMSO controls (7.3 ± 0.7 vs. 5.3 ± 1.1 μm, *p* = 0.002, *t*_(12)_ = 3.94, unpaired *t*-test, *n* = 7 utricles per condition, Figures 3C–E), corresponding to a 24% decrease in elastic modulus (6.5 ± 1.1 vs. 4.6 ± 0.5 kPa, *p* = 0.002, *t*_(12)_ = 3.91, unpaired *t*-test, *n* = 7 utricles per condition, Figure 3F). Thus, F-actin substantially stiffens the utricular epithelium in adult mice.

Next, we investigated the expression of actin-binding genes in mouse utricles using RT-qPCR. We observed that *Alpha-Actinin-4* (*Actn4*), which cross-links actin filaments and tethers them to cadherin-catenin complexes at adherens junctions (Tang and Brieher, 2012), increases 2.4-fold from P0 to P83 (*p* < 0.0001, *t*_(4)_ = 18.3, *n* = 3 replicates, Supplementary Figure 2A). Consistent with this, examination of publicly available RNA sequencing data of mouse utricular sensory epithelia revealed a 1.4-fold increase in *Actn4* expression between E17 and P9 (*p* = 0.0114, *t*_(4)_ = 4.43, *n* = 3 replicates, Supplementary Figure 2B; Gnedeva and Hudspeth, 2015). We detected the ortholog *ACTN4* in chicken utricles by RT-qPCR but found no significant change in expression between the ages of P0 and P30-60 (*p* = 0.28, *t*_(2)_ = 1.46, *n* = 2 replicates, Supplementary Figure 2A).

We observed that Actn4 localizes to the exceptionally thick circumferential F-actin bands in mouse SCs (Figures 3G,I). To characterize the effect of *Actn4* on the morphology of the F-actin bands, we used *Actn4* KO mice that have whole-body deletion of the *Actn4* gene (Kos et al., 2003). Since mice with homozygous deletion of *Actn4* rarely survived past weaning age (21/175, 12%), we obtained TEM images from utricles of P23 *Actn4* KO and wild-type mice. Deletion of *Actn4* reduced the density of the perijunctional F-actin network as can be seen in Supplementary Figure 3.

To circumvent the perinatal lethality of whole-body *Actn4* deletion, we generated conditional knockout mice in which *Actn4* deletion is restricted to the embryonic forebrain and other discrete head structures, including the otic vesicle (*Actn4* cKO, see section “Materials and Methods”). *Actn4* cKO mice were viable and fertile, and showed no significant difference in the area of the utricular sensory epithelia compared to *Actn4^flox/flox^* littermate controls (1098 ± 62 vs. 1126 ± 85 μm^2^, *p* = 0.43, *t*_(6)_ = 0.85, unpaired *t*-test, *n* = 4 adult mice per condition, Supplementary Figure 4). Conditional deletion of *Actn4* in vestibular SCs was confirmed by immunostaining, which showed that morphology of the F-actin bands was disrupted in utricles where the cross-linker was absent (Figures 3H,J).

To test whether *Actn4* cross-linking within the F-actin bands stiffens the utricular epithelium, we performed micropipette aspiration on utricles from aged (16–24 months) *Actn4* cKO mice and littermate controls. The epithelial surface of the utricles from *Actn4* cKO mice deformed 19% more than *Actn4^flox/flox^* littermate controls (5.4 ± 0.4 vs. 4.5 ± 0.6 μm, *p* = 0.012, *t*_(18)_ = 2.79, unpaired *t*-test, *n* = 10 utricles per condition, Figure 3K). That corresponds to an 18% reduction in elastic modulus (6.4 ± 0.4 vs. 7.6 ± 0.8 kPa, *p* = 0.005, *t*_(18)_ = 3.22, unpaired *t*-test, *n* = 10 utricles per condition, Figure 3L). The results thus far showed that the mouse utricular epithelium stiffens as it matures, that the apical junctions are sites of particular stiffness, and that stiffness depends on both the F-actin bands and Alpha-Actinin-4. Contrasting with that, the F-actin belts of SCs in the chicken utricular epithelium remain thin, readily regenerate HCs even as adults, and are considerably more compliant than their counterparts in mice.

### Supporting Cell Elongation in the Chicken Utricle Provides Evidence of Anisotropic Tissue Tension

The shapes of epithelial cells reflect a balance of forces from internal hydrostatic pressure, adhesion, and actomyosin contractility (Mao and Baum, 2015). In the course of this investigation, we observed that SC surfaces in wild-type mice resemble regular polygons, while those in chickens are elongated (Supplementary Figures 5A,E). To determine whether and how differences in cell surface shapes arose during development, we measured the outlines of SC surfaces at multiple developmental timepoints, quantifying changes in SC area, elongation, and alignment (Supplementary Figures 5A–I).

**FIGURE 4:**
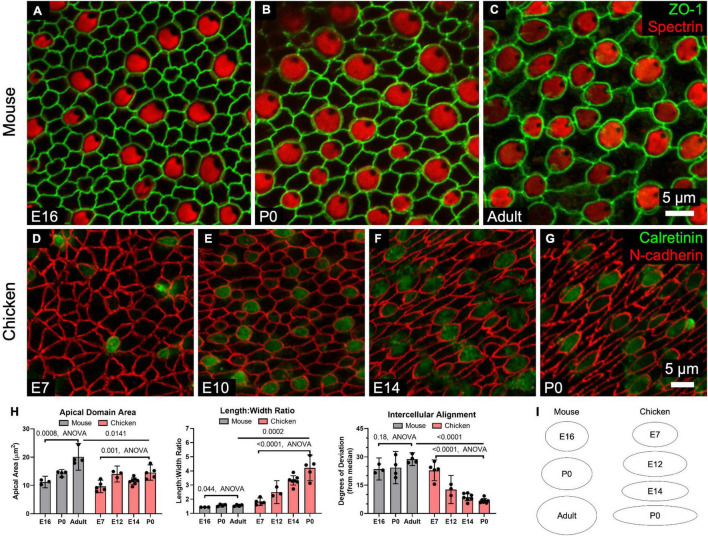
SCs in mouse utricles do not undergo dramatic shape changes during development and postnatal maturation, whereas those in chickens become elongated and locally aligned during development. **(A–C)** Confocal images of utricles from mice of various ages. Images are from the striolar region. **(D–G)** Confocal images of utricles from chickens of various ages. **(H)** Quantification reveals that SC apical domains become significantly larger over time (Mouse: *p* = 0.0008, *F*_(2,8)_ = 20.0, ANOVA, *n* = 3–4 utricles per condition; Chicken: *p* = 0.001, *F*_(3,16)_ = 9.05, ANOVA, *n* = 3–7 utricles per condition). The mean SC area was significantly greater in adult mice than P0 chickens (*p* = 0.0141, *t*_(7)_ = 3.2, unpaired *t*-test, *n* = 4–5 utricles per condition). Both mouse and chicken SCs became significantly more elongated with age (Mouse: *p* = 0.044, *F*_(2,8)_ = 4.7, ANOVA, *n* = 3–4 utricles per condition; Chicken: *p* = 0.001, *F*_(3,16)_ = 25.5, ANOVA, *n* = 3–7 utricles per condition), though elongation was significantly greater in P0 chicken SCs compared to SCs in adult mice (*p* = 0.0002, *t*_(7)_ = 7.1, unpaired *t*-test, *n* = 4–5 utricles per condition). Alignment did not significantly change in mouse SCs with age (*p* = 0.18, *F*_(2,8)_ = 2.1, ANOVA, *n* = 3–4 utricles per conditions), but significantly increased with age in chicken SCs (*p* < 0.0001, *F*_(3,18)_ = 39.9, ANOVA, *n* = 3–7 utricles per condition). SCs of the P0 chicken utricle were significantly more aligned than those of adult mouse utricles (*p* < 0.0001, *t*_(9)_ = 21.9, unpaired *t*-test, *n* = 4–7 utricles per condition). **(I)** The average shapes of SC apical domains in mouse and chicken utricles are depicted as ellipses to denote relative area and length:width ratio.

In mouse utricles, the area enclosed by the surface outline of the average SC increased by ∼80% from E16 through adulthood (*p* = 0.0008, *F*_(2,8)_ = 20.0, ANOVA, *n* = 3–4 utricles per condition, Figures 4A–C,H,I). The average area of chicken SCs also grew with developmental age, increasing ∼50% from E7 to P0 (*p* = 0.001, *F*_(3,16)_ = 9.05, ANOVA, *n* = 3–7 utricles per condition, Figures 4D–I).

Differences in surface structure between mouse and chicken SCs became more apparent when we assessed elongation. The surfaces of mouse SCs maintained fairly regular shapes, with the length:width ratios of cell outlines increasing by just ∼8% between E16 and adulthood (E16: 1.4 ± 0.01, Adult: 1.6 ± 0.1, *p* = 0.044, *F*_(2,8)_ = 4.7, ANOVA, *n* = 3–4 utricles per condition, Figure 4H). In contrast, SC surfaces in embryonically developing chickens became progressively elongated. By P0, the length:width ratio of chicken SCs averaged 4.2 ± 0.9, more than twice the ratio for SCs at E7 (*p* = 0.001, *F*_(3,16)_ = 25.5, ANOVA, *n* = 3–7 utricles per condition, Figure 4H).

By observing the orientation of HC cuticular plates, which we immunostained using an antibody to spectrin, we determined the local axis of planar HC polarity. Immunostaining for spectrin and occludin revealed that chicken SCs are elongated and aligned parallel to the local axis of planar HC polarity (Supplementary Figure 5J). At E7, the deviation of the average SC from the local median angle was 23° ± 6° but the average deviation shrank to just 7° ± 1° by P0 (*p* < 0.0001, *F*_(3,18)_ = 39.9, ANOVA, *n* = 3–7 utricles per condition, Figure 4H). In contrast, mouse SCs did not exhibit detectable alignment with age (E16: 24° ± 6°, Adult: 24° ± 9°, *p* = 0.18, *F*_(2,8)_ = 2.1, ANOVA, *n* = 3–4 utricles per condition, Figure 4H).

The measurements show that the regular polygonal apical surfaces of SCs in mouse utricles approximate a standard, low-energy configuration. In contrast, SCs in the developing chicken utricle become elongated and collectively aligned with the planar polarity axis, which is a higher-energy configuration and likely results from anisotropic forces in the epithelium.

### Normal Supporting Cell Shape Requires Maintained Actomyosin Contractility in the Utricles of Chickens, but Not in Mice

**FIGURE 5:**
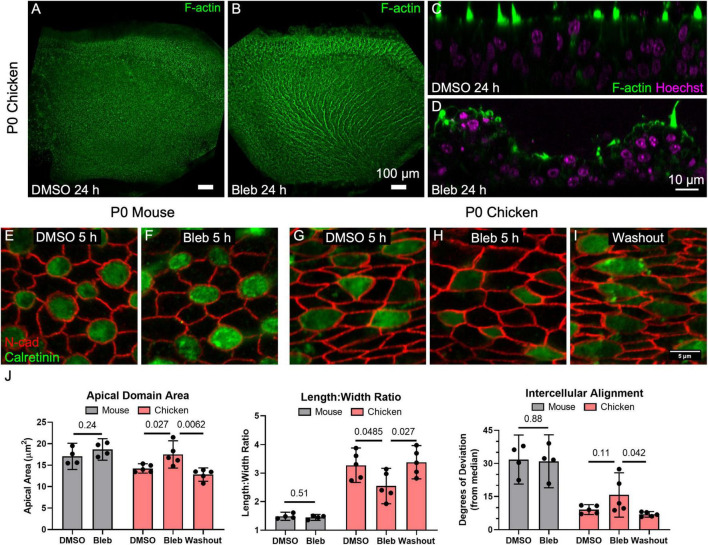
Inhibition of non-muscle myosin II activity led to dramatic expansion of SC apical domains in utricles of P0 chickens but had no detectable effect on the shapes of SCs in P0 mouse utricles. **(A,B)** Chicken utricles were cultured for 24 h in the presence of DMSO **(A)** or 50 μm blebbistatin **(B)**. Blebbistatin treatment resulted in a “ruffled” appearance of the epithelial surface. **(C,D)** Z-projections depicting buckling of the epithelium after blebbistatin treatment. **(E–I)** Utricles from P0 mice and P0 chickens were cultured for 5 h in the presence of 50 μM blebbistatin or DMSO vehicle control before fixation. Some blebbistatin-treated utricles of P0 chickens were rinsed and fixed after a total of 16 h (Washout, **I**). **(J)** Quantification revealed that in SCs of mouse utricles, blebbistatin treatment did not lead to significant differences in apical domain area (*p* = 0.24, *t*_(6)_ = 1.30, unpaired *t*-test, *n* = 4 utricles per condition), length:width ratio (*p* = 0.51, *t*_(6)_ = 0.70, unpaired *t*-test, *n* = 4 utricles per condition), or intercellular alignment (*p* = 0.88, *t*_(6)_ = 0.15, unpaired *t*-test, *n* = 4 utricles per condition) compared to controls treated with DMSO. In contrast, blebbistatin treatment significantly affected SC area (*p* = 0.027, *t*_(8)_ = 2.7, *n* = 5 utricles per condition) and length:width ratio (*p* = 0.0485, *t*_(8)_ = 2.3, *n* = 5 utricles per condition) in chicken utricles, with alignment not reaching statistical significance (*p* = 0.11, *t*_(8)_ = 1.8, *n* = 5 utricles per condition). Upon washout of blebbistatin, changes to SC area (*p* = 0.0062, *t*_(8)_ = 3.7, *n* = 5 utricles per condition), elongation (*p* = 0.027, *t*_(8)_ = 2.3, *n* = 5 utricles per condition), and intercellular alignment (*p* = 0.042, *t*_(8)_ = 2.7, *n* = 5 utricles per condition) were reversible.

The contractile force of non-muscle myosin II on the junction-associated F-actin belt is an important determinant of cell shape in epithelia (Ebrahim et al., 2013). Mouse and chicken utricles both express non-muscle myosin II isoforms as illustrated by RNA-sequencing data (Supplementary Figure 2C; Ku et al., 2014; Gnedeva and Hudspeth, 2015). We hypothesized that myosin II contractility influences the shape of vestibular SC surfaces. To test this, we cultured P0 chicken utricles in the presence of either DMSO or 50 μM blebbistatin, an inhibitor of non-muscle myosin II contractility, for 24 h before fixing and immunostaining for N-cadherin and calretinin to label cell junctions and HCs, respectively. Confocal microscopy showed a normal, approximately planar sensory epithelium surface in each of the DMSO-treated utricles, but the sensory epithelium in each of the utricles cultured in blebbistatin developed a striking series of ridges and valleys, which we refer to as “ruffles” (Figures 5A,B). The ruffles were regularly spaced ∼25 μm apart and fanned out laterally, paralleling the local axis of HC polarity. *Z*-axis projections from confocal microscopy revealed that the epithelial surface of the blebbistatin-treated utricles had buckled (Figures 5C,D).

To determine how this dramatic change in sensory epithelium form came about, we recorded images from P0 chicken utricles in time-lapse microscopy after labeling with SiR-actin, a cell-permeable live-cell probe that is highly specific for F-actin. At the start of image acquisition, DMSO was spiked into the medium containing a chicken utricle in one half of a two-compartment imaging chamber, and blebbistatin was spiked into the medium around another chicken utricle in the other chamber at a final concentration of 50 μM. The shapes of SCs in the DMSO-treated utricles did not change during the 9 h time-lapse (Supplementary Movie 3), while the apical surfaces of SCs in the blebbistatin-treated utricles began to expand noticeably starting at ∼3 h. We expected that shape changes would occur stochastically, but instead the time-lapse recordings revealed SC surfaces changing shape and size in concert with their neighbors. When the time-lapse images encompassed larger areas of the sensory epithelium it became apparent that the cellular surface expansion was propagating across the epithelium, suggesting a wavefront of SC surface relaxation. Measurements of SC surface outlines showed that the expansion of SCs was anisotropic, with the short axis of the SC surfaces increasing 1.1 ± 0.2 μm on average, which was more than three times the 0.3 ± 0.5 μm average increase in the long axis (*p* = 0.006, *t*_(29)_ = 3.0, paired *t*-test, *n* = 30 cells). We conclude that a previously unrecognized requirement for actomyosin contractility maintains anisotropic tension and normal supporting cell surface shapes in the chicken utricular epithelium, since inhibition of myosin II contractility quickly led to anisotropic SC expansion that resulted in buckling of the sensory epithelium.

To further quantify the effect, we cultured utricles from chickens and mice in the presence of DMSO or 50 μM blebbistatin for 5 h. At that point, most cultures were either fixed and processed for immunohistochemistry, but some blebbistatin-treated utricles were cultured for 16 h before rinsing and fixation. The 5-h blebbistatin treatment of P0 chicken utricles resulted in a 20% increase in SC area and a 20% decrease in the average SC length:width ratio (area: *p* = 0.027, *t*_(8)_ = 2.7; length:width ratio: *p* = 0.0485, *t*_(8)_ = 2.3; alignment: *p* = 0.11, *t*_(8)_ = 1.8, unpaired *t*-test, *n* = 5 utricles per condition, Figures 5G–J). Upon washout, the effects of blebbistatin on SCs in the chicken utricle were reversible (area: *p* = 0.0062, *t*_(8)_ = 3.7; length:width ratio: *p* = 0.027, *t*_(8)_ = 2.3; alignment: *p* = 0.042, *t*_(8)_ = 2.7, unpaired *t*-test, *n* = 5 utricles per condition, Figures 5G–J).

In contrast to the dramatic effects we observed in the chicken utricle, blebbistatin treatments of P0 mouse utricles produced no detectable differences in SC area, elongation, or alignment (area: *p* = 0.24, *t*_(6)_ = 1.30; length:width ratio: *p* = 0.51, *t*_(6)_ = 0.70; alignment: *p* = 0.88, *t*_(6)_ = 0.15, unpaired *t*-test, *n* = 4 utricles per condition, Figures 5E,F,J). We repeatedly tested this in our laboratory but have not found any experimental conditions under which blebbistatin inhibition of myosin II in mouse utricles causes substantial changes in SC shape or leads to epithelial buckling.

Since chickens are precocious at hatching and newborn mice are altricial, P0 chicken utricles may be at a more advanced developmental stage than P0 mouse utricles. For that reason, we repeated the experiment using blebbistatin treatment of P75 mouse utricles, which yielded no detectable differences in SC area or intercellular alignment (area: *p* = 0.46, *t*_(6)_ = 0.80; alignment: *p* = 0.11, *t*_(6)_ = 1.90, *n* = 4 utricles), and a minimal 5% reduction in length:width ratio (*p* = 0.031, *t*_(6)_ = 2.80, *n* = 4 utricles, Supplementary Figure 6). The results show that the sizes and shapes of F-actin-stiffened apical domains of SCs in mouse utricles are maintained independent of non-muscle myosin II contractility. In sharp contrast, the more readily deformable SCs in the chicken utricle require continuous actomyosin contractility to maintain their distinct elongated and aligned surface shapes.

### Supporting Cells in Chicken Utricles Divide Perpendicular to Hair Cell Polarity During Development and Regeneration

**FIGURE 6:**
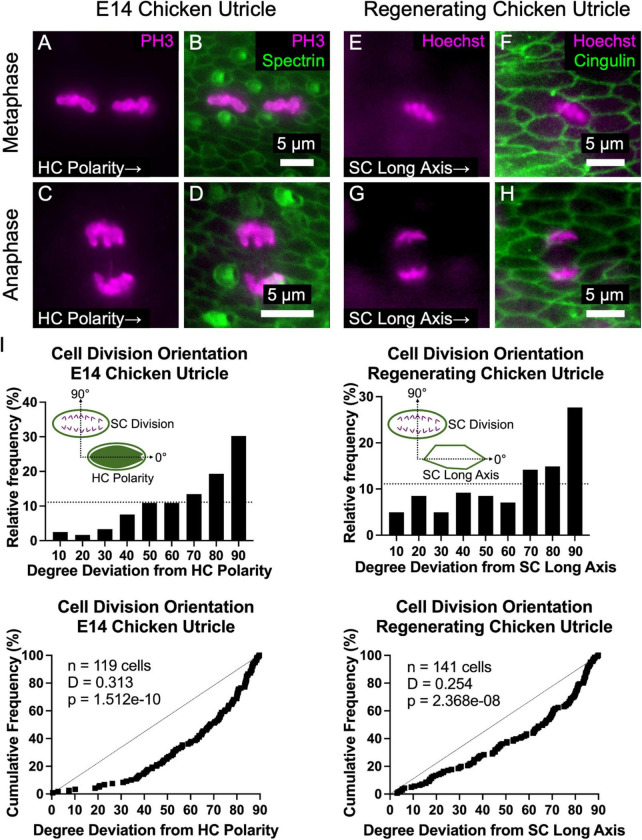
The majority of supporting cell divisions in the developing and regenerating chicken utricle are oriented such that the division axis is perpendicular to the axes of HC polarity and SC elongation. **(A–D)** The orientation of PH3-labeled mitotic figures in metaphase **(A,B)** and anaphase **(C,D)** were measured with respect to the local axis of HC polarity, as visualized by spectrin immunostaining. **(E–H)** To determine whether regenerative cell divisions were oriented, utricles from P2 chickens were cultured in 1 mM streptomycin for 24 h to ablate HCs and fixed after a total of 72 h. Due to the high efficiency of HC killing with this approach, no HCs are present in the images. Images of cells in metaphase **(E,F)** and anaphase **(G,H)** were acquired with Hoechst labeling of cell nuclei, and the orientation of mitotic figures was measured with respect to the local axis of SC elongation, as visualized by cingulin immunostaining. **(I)** *Top:* Histograms of cell division orientation during development and regeneration. The bins have a width of 10 degrees and the *x*-axis labels denote the upper boundary of each bin. The dashed lines denote a uniform distribution (i.e., randomly oriented divisions). *Bottom:* Cumulative distribution functions of cell division orientation during development and regeneration. Dashed lines denote randomly oriented cell divisions. KS tests revealed that the measured cell division orientations were significantly non-random during development (*p* = 1.512e-10, *D* = 0.313, *n* = 119 cells) and regeneration (*p* = 2.368e-8, *D* = 0.254, *n* = 141 cells).

In epithelial monolayers that experience anisotropic tensional stress, cell divisions typically occur with the cleavage plane orthogonal to the cell’s long axis, so the division of an elongate cell results in two less elongated cells and reduced tensional stress (Wyatt et al., 2015). The propensity for elongated cells to divide along that axis is known as “Hertwig’s rule” (Hertwig, 1884). To determine whether the cell divisions of elongated SCs in the chicken utricle are oriented in a non-random manner, we harvested utricles at E14 and immunostained them with anti-phospho-Histone 3 (PH3), which binds to condensed chromatin during mitosis. The orientations of 119 cells that were in metaphase or anaphase were analyzed with reference to the local axis of HC polarity, as determined by spectrin immunostaining (Figures 6A–D). The measured sample of cell division orientations was clearly non-random (*p* = 1.512e^–10^, *D* = 0.313, KS test, *n* = 119 cells, Figure 6I). We were surprised to see that the majority of mitotic figures were oriented orthogonal to the planar axis of HC polarity, so the cleavage plane of the ensuing cell divisions would occur parallel to the SC’s long axis in violation of “Hertwig’s rule” (Figures 6A,B).

To test whether regenerative cell divisions orient similarly, we harvested utricles from P2 chickens and killed HCs by culturing the utricles in the presence of 1 mM streptomycin for 24 h, prior to washout and culturing 48 h more in control medium before fixation. Orientations of 141 mitotic figures in metaphase and anaphase were measured relative to the local axis of SC elongation, which is aligned with the axis of HC polarity (Supplementary Figure 5J). As in development, regenerative cell divisions were not randomly oriented (*p* = 2.368e^–8^, *D* = 0.254, KS test, *n* = 141 cells, Figure 6I). Rather, the majority of mitotic figures were oriented such that the cleavage plane would be parallel to the SC’s long axis (Figures 6E–I). Thus, in development and regeneration, and contrary to “Hertwig’s rule,” cell divisions in chicken utricles tend to orient in a manner that would appear to contribute to, rather than dissipate, anisotropic tensional stress.

## Discussion

Auditory SCs in mammals develop highly specialized morphologies and are positioned in precisely ordered rows along the organ of Corti, making them significantly different from their less specialized counterparts in the auditory epithelia of fish, amphibians, reptiles, and birds. SCs in the vestibular organs of humans and other mammals closely resemble those of non-mammals in overall form and positioning, but they lack the high capacity for regenerative replacement of lost HCs that allows non-mammals to quickly recover sensory function. There are, however, two cellular specializations that distinguish vestibular SCs in mammals from those in nearly all non-mammals: The first to be recognized in mammalian SCs was the exceptionally robust circumferential F-actin bands, which grow in thickness until they occupy 89% of the average SC area at the level of the adherens junction (Meyers and Corwin, 2007; Burns et al., 2008). The second recognized specialization was E-cadherin, which is expressed at high levels in the adherens junctions and basolateral membranes of mammalian SCs but is absent or expressed at very low levels in the SCs and vestibular epithelia of birds, turtles, amphibians, bony fish, and sharks (Collado et al., 2011b; Burns et al., 2013). Those observations and the results of prior experiments led to two hypotheses: that the highly reinforced, E-cadherin-rich junctions in mature mammalian vestibular epithelia might dampen local intraepithelial tension changes that arise when cells are lost, and that such dampening of those mechanical signals might reduce the capacity for mammalian SCs to respond to HC losses by changing shape and participating in regenerative proliferation (Meyers and Corwin, 2007; Burns and Corwin, 2013).

Prior to this study, there was a lack of direct evidence as to whether the reinforced adherens junctions of mammalian SCs affect the mechanical properties of the utricular epithelium. Our results show that accumulation of F-actin and the expression of Alpha-Actinin-4 at SC-SC junctions stiffen the utricular epithelium substantially as mice mature. Stiffness measured in the mouse sensory epithelium was considerably greater than our measurements from the highly regenerative chicken utricle, where the F-actin belts remain thin throughout life. While blebbistatin-mediated inhibition of non-muscle myosin II contractility produced no detectable changes in the shape or surface area of SCs in mouse utricles, in chicken utricles it caused SC shape change, dramatic expansion of SC surfaces, and buckling of the sensory epithelium. Taken together, our findings show that the utricular sensory epithelium in chickens behaves as a mechanical syncytium, with deformable SCs collectively responding to changes in intraepithelial tension such as those produced when cells are lost from the epithelium. Epithelial cell loss deforms nearby cells, changing tension in their cortical F-actin and intercellular junctions (Karsch et al., 2017), which can activate mitogenic YAP-TEAD signaling (Rauskolb et al., 2014; Benham-Pyle et al., 2015). The mechanical differences measured in this study are consistent with unexplained species differences in SC responses and HC regeneration. When HCs in chicken ears die, nearby, compliant SCs rapidly change shape, which is associated with robust nuclear accumulation of YAP and subsequent S-phase entry (Cotanche, 1987; Corwin and Cotanche, 1988; Bird et al., 2010; Collado et al., 2011a; Rudolf et al., 2020).

In contrast with the properties of utricular epithelium in chickens, the mechanical properties of the utricular sensory epithelium in the mouse are dominated by stiffness that depends in part on exceptionally thick and cross-linked F-actin bands at the level of SC junctions. It appears likely that the apical domain stiffness of mammalian supporting cells limits and blunts dynamic mechanical signals produced during cell loss and restricts collective mechanical behavior that can govern more effective epithelial repair. Consistent with this, the progressive stiffening of adherens junctions in mouse SCs correlates with reduced rates of shape change and proliferation, and with restricted responses to HC loss observed by time-lapse microscopy that are limited to just the closest neighboring SCs (Meyers and Corwin, 2007; Collado et al., 2011a; Burns and Corwin, 2014).

In another study, we recently reported that treatments with EGF and a GSK3β inhibitor cause significant thinning of the circumferential F-actin bands in SCs throughout the sensory epithelium in utricles cultured from adult mice (Kozlowski et al., 2020). With time, treatment with EGF and a GSK3β inhibitor caused depletion of E-cadherin at the junctions between SCs located within the striola and resulted in significant SC proliferation that was restricted to the striola. It is noteworthy that thinning of F-actin bands occurred throughout the entire sensory epithelium in those adult utricles but was not in itself sufficient to trigger proliferation. It remains to be determined whether thinning of the F-actin is required for E-cadherin depletion at SC-SC junctions in adult mouse utricles and why the effect on E-cadherin levels was restricted to just the striolar SCs. Our data suggest that treatments with EGF and a GSK3β inhibitor would reduce the stiffness of SCs in the adult mouse utricle, but it remains unknown whether that would cause those SCs to be more responsive to inhibition of myosin II contractility or better able to activate YAP-TEAD signaling. Further studies are required to investigate whether mouse models that have enhanced regenerative capacity have SCs with thinner F-actin bands, greater compliance, and heightened YAP-TEAD signaling.

We did not identify mechanisms that underlie the mouse utricle’s lack of sensitivity to blebbistatin inhibition of myosin II contractility (Figure 5). One possibility is that presence of Actn4 or other actin cross-linkers such as Filamin A and Tropomodulin 1, which also localize to the F-actin bands (data not shown), could reduce the efficiency of actomyosin contractility (Ennomani et al., 2016). Interestingly, blebbistatin treatments are reported to induce expansion of SC surfaces in cochleae explanted from embryonic and newborn mice (Ebrahim et al., 2013; Cohen et al., 2020), where circumferential F-actin bands are not as thick as in utricular SCs (Burns et al., 2008, 2013). The thinner F-actin bands in SCs of the developing cochlea may be important for convergent extension, cell intercalation, and other morphogenic movements that are essential to the development of the organ of Corti’s precise patterning (Cohen et al., 2020). The circumferential F-actin bands of Deiters cells and pillar cells thicken postnatally (Burns et al., 2013), and the epithelial surface becomes stiffer (Szarama et al., 2012; Chen et al., 2021), but it remains to be determined whether SCs in the cochleae of older mice become insensitive to blebbistatin treatment.

The findings presented here suggest that the thick circumferential F-actin bands could have provided two selective advantages during mammalian evolution. Stiffness conferred by the thick F-actin bands may have contributed to enhanced fidelity of HC mechanoelectrical transduction, particularly at high sound frequencies where the sensitivity of numerous mammalian species greatly exceeds that in birds and other non-mammals. The thick F-actin bands of mammalian SCs also appear likely to have imparted an energetic advantage, since the maintenance of mammalian SC shape does not rely on constitutively maintained, ATP-dependent activity of non-muscle myosin II, as shown in the blebbistatin results. In contrast, SCs of the utricles of birds appear to require continuous expenditure of ATP to sustain myosin II contractility that maintains their compact apical domains, surface shapes and sizes, as well as the planar form of the chicken utricular epithelium, which changes dramatically when myosin II contraction is inhibited. Both potential advantages warrant further exploration, as does the possibility that the mechanical properties and responses of SCs in chicken vestibular epithelia may be shared with the vestibular epithelia in reptiles, amphibians, bony fish, and sharks that also retain thin circumferential F-actin belts throughout life and express little or no E-cadherin in the sensory epithelium (Burns et al., 2013).

The progressive elongation and alignment of SCs in the embryonic chicken utricle is a hallmark of accumulating anisotropic tensile stress. Typically, cell divisions are oriented such that the cleavage plane occurs perpendicular to the cell’s long axis (Hertwig, 1884), which reduces anisotropic stress (Campinho et al., 2013; Wyatt et al., 2015). Yet, contrary to “Hertwig’s rule,” during development and in regeneration SC mitoses in the chicken utricle are oriented such that the cleavage will be parallel to the cell’s long axis (Figure 6). The results suggest that the cell divisions and subsequent cell growth would increase anisotropic stress. Time-lapse microscopy and other measures will be needed to confirm or refute that hypothesis. An intriguing possibility is that the orientation of the SC divisions promotes the accumulation and maintenance of tissue anisotropic stress that could contribute to the planar polarization or the refinement of HC orientation.

While the downregulation of developmental pathways such as Wnt, Notch, and SoxC has appropriately garnered attention in efforts to explain the reduced regenerative capacity of mammalian HC epithelia (Gnedeva and Hudspeth, 2015; Maass et al., 2015; Wang et al., 2015), the measurements and experimental findings reported above suggest that it would be wise to more fully investigate the unique cytological features and expression patterns that arose in SCs as mammals evolved. The results here sharpen mechanistic understanding of how the junctional reinforcement in mammalian SCs may limit regenerative responses, with exceptionally thick circumferential F-actin bands providing stiffness and the potential to act as a “biomechanical brake” that limits deformations, actomyosin-generated tension, and the propagation of tension changes in mammalian HC epithelia. High levels of E-cadherin may not only provide strong intercellular adhesion, but may collaborate as a “biochemical brake” that sequesters mitogenic signaling molecules at adherens junctions, as recent evidence suggests (Kozlowski et al., 2020). These mechanisms may explain, in part, why mammalian SCs largely remain in a state of proliferative quiescence after HC loss, whereas SCs in non-mammals readily replace HCs, enabling recovery of hearing and balance function.

## Data Availability Statement

The raw data supporting the conclusions of this article will be made available by the authors, without undue reservation.

## Ethics Statement

The animal studies were reviewed and approved by the Animal Care and Use Committee at the University of Virginia (protocol 18350718) and National Institutes of Health guidelines for animal use (protocol 1254-18).

## Author Contributions

MR, AA, AC-R, and JC designed the research and edited the manuscript. MR, AA, CK, AD, AK, WB, and AC-R performed the research. MR, CK, AD, AK, WB, and AC-R analyzed the data. MR wrote the first draft of the manuscript. All authors contributed to the manuscript revision, read and approved the submitted version.

## Conflict of Interest

The authors declare that the research was conducted in the absence of any commercial or financial relationships that could be construed as a potential conflict of interest.

## Publisher’s Note

All claims expressed in this article are solely those of the authors and do not necessarily represent those of their affiliated organizations, or those of the publisher, the editors and the reviewers. Any product that may be evaluated in this article, or claim that may be made by its manufacturer, is not guaranteed or endorsed by the publisher.
