# Supplementary material for: Stiffening of Circumferential F-Actin Bands Correlates With Regenerative Failure and May Act as a Biomechanical Brake in the Mammalian Inner Ear
Source: Front Cell Neurosci. 2022 May 4;16:859882. doi: 10.3389/fncel.2022.859882 (PMC9114303; doi:10.3389/fncel.2022.859882)
Supplement: Supplementary file 1 [file Data_Sheet_1.docx]

Supplementary Material

# Supplementary Figures


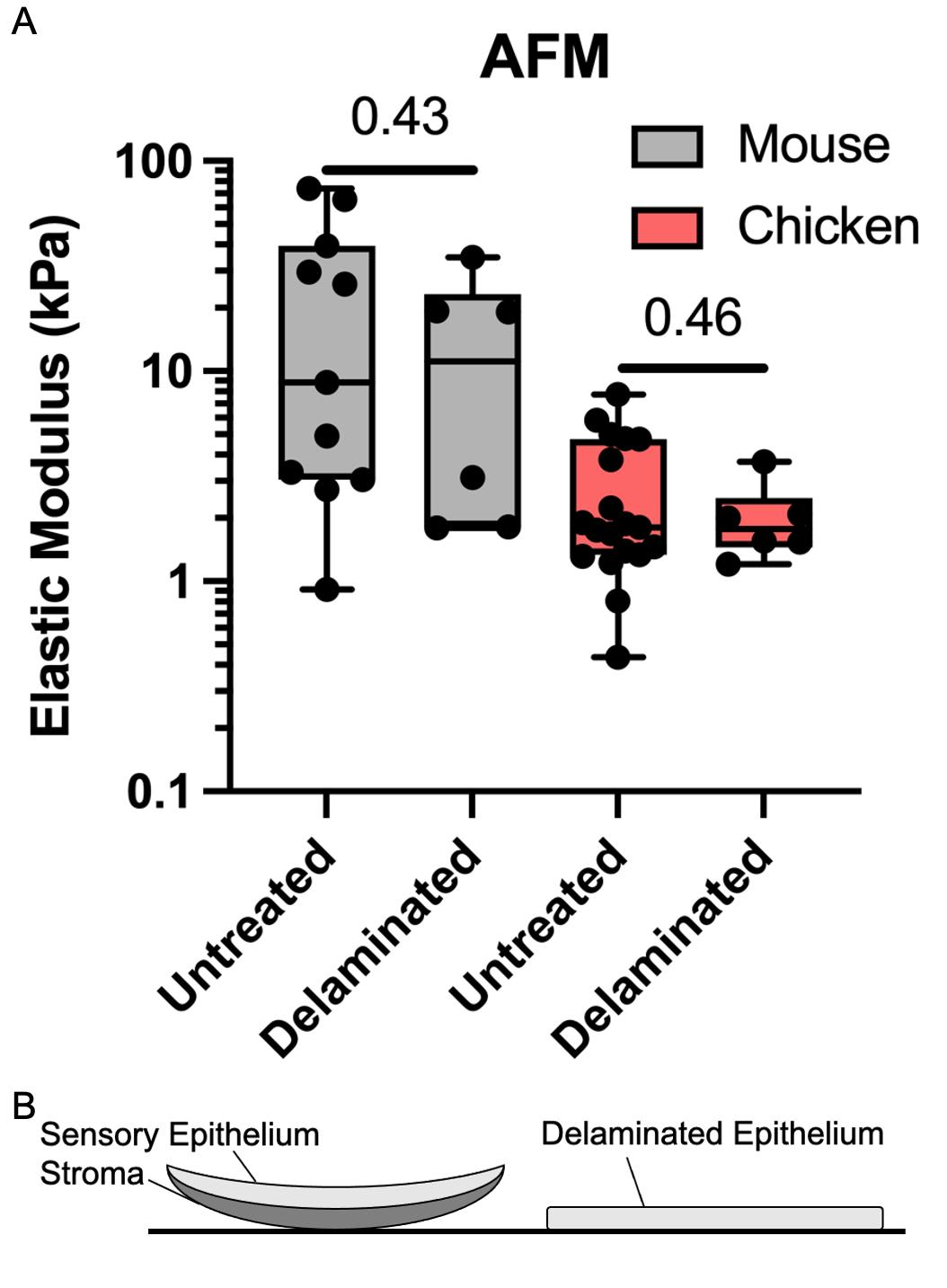


**Supplementary Figure 1.** **(A)** AFM measurements of utricular epithelia from adult mice and embryonic chickens are unaffected by the presence of underlying stroma. Dots represent the average elastic modulus obtained from an eight-point line scan. The data are plotted on a logarithmic scale. P-values are indicated from Mann-Whitney tests. **(B)** Schematic of untreated and delaminated utricles. Untreated utricles contain sensory epithelium and underlying stroma. In the delaminated epithelial sheets, the underlying stroma is enzymatically and mechanically removed.


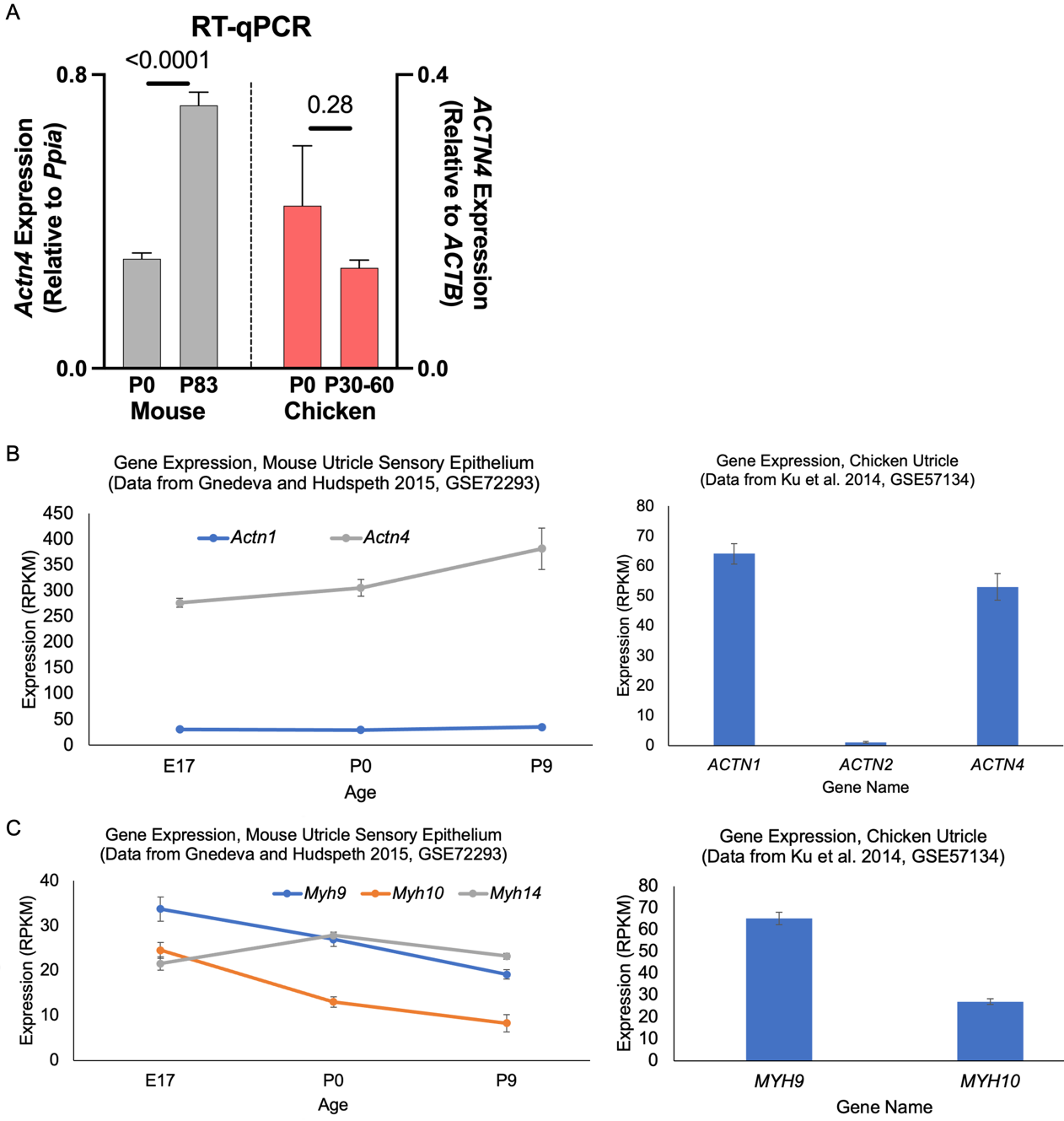


**Supplementary Figure 2.** **(A)** RT-qPCR data depicting *Alpha-actinin-4* expression in mouse and chicken utricles. Levels of mouse *Actn4* increase 2.4-fold between P0 and P83 (p < 0.0001, t_(4)_ = 18.3, n = 3 biological replicates). No significant difference in expression of chicken *ACTN4* was detected between P0 and P30-60 (p = 0.28, t_(2)_ = 1.46, n = 2 biological replicates). **(B)** Publicly available RNA-sequencing data for alpha actinins in mouse utricular sensory epithelium (Gnedeva and Hudspeth 2015, GSE72293) and chicken utricles (Ku et al., 2014; GSE57134). The expression of mouse Actn4 increases 1.4-fold between E17 and P9 (p = 0.0114, t_(4)_ = 4.43, n = 3 biological replicates). The genes *Actn2* and *Actn3* were not detected above threshold levels (>5 RKPM) in mouse utricle sensory epithelia, and there is no *Actn3* ortholog in chicken. **(C)** Publicly available RNA-sequencing data for non-muscle myosin II isoforms in mouse utricular sensory epithelium (Gnedeva and Hudspeth 2015; GSE72293) and chicken utricles (Ku et al., 2014; GSE57134). *Myh9*, *Myh10*, and *Myh14* encode the A, B, and C isoforms of non-muscle myosin II, respectively. There is no chicken ortholog for *Myh14*. Graphs depict mean ± standard deviation.


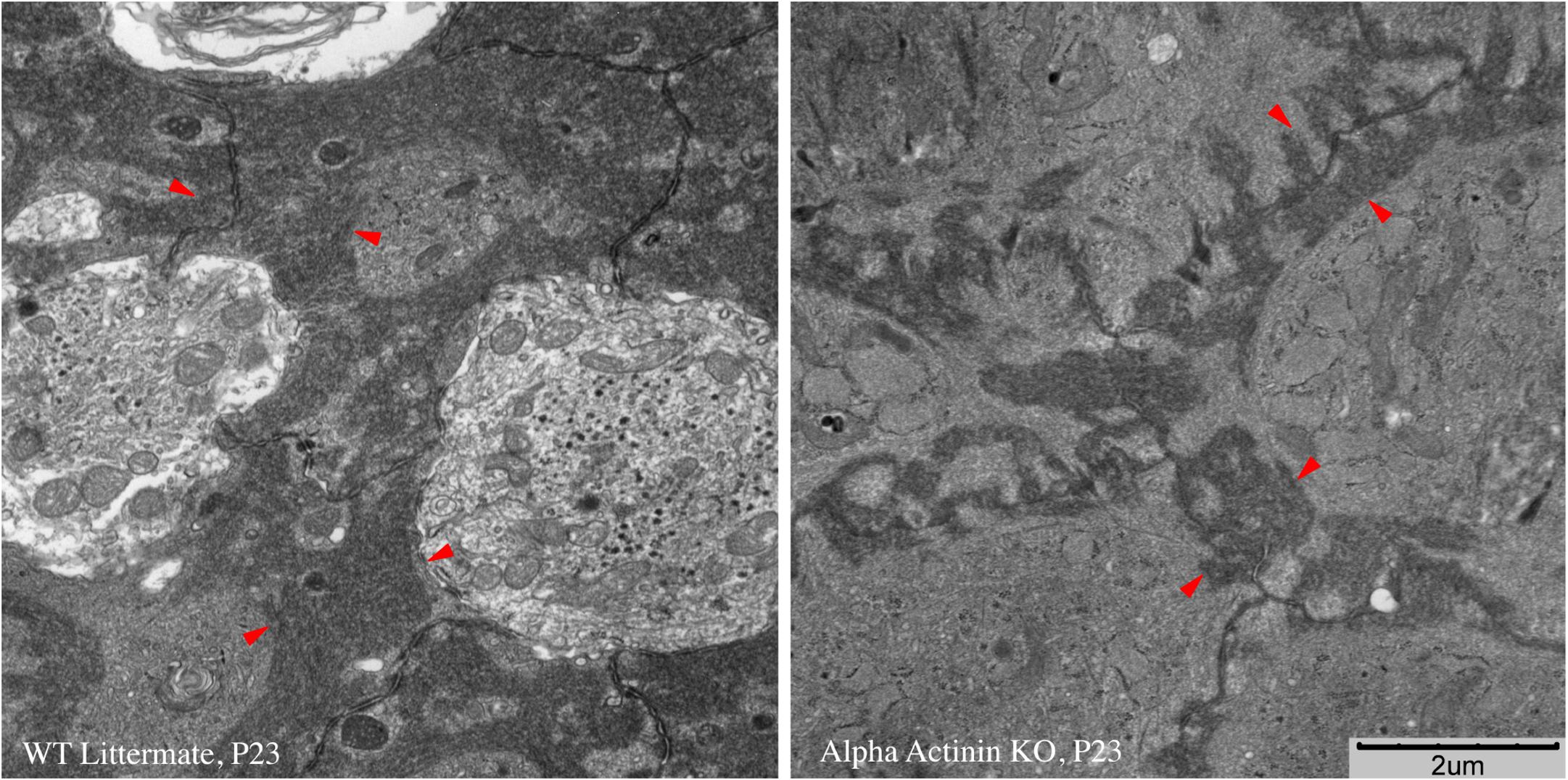


**Supplementary Figure 3.** TEM images of *Actn4* KO mice and littermate controls reveal that Actn4 deletion reduces the density of the perijunctional F-actin network. The red arrowheads bracket the electron-dense F-actin network.


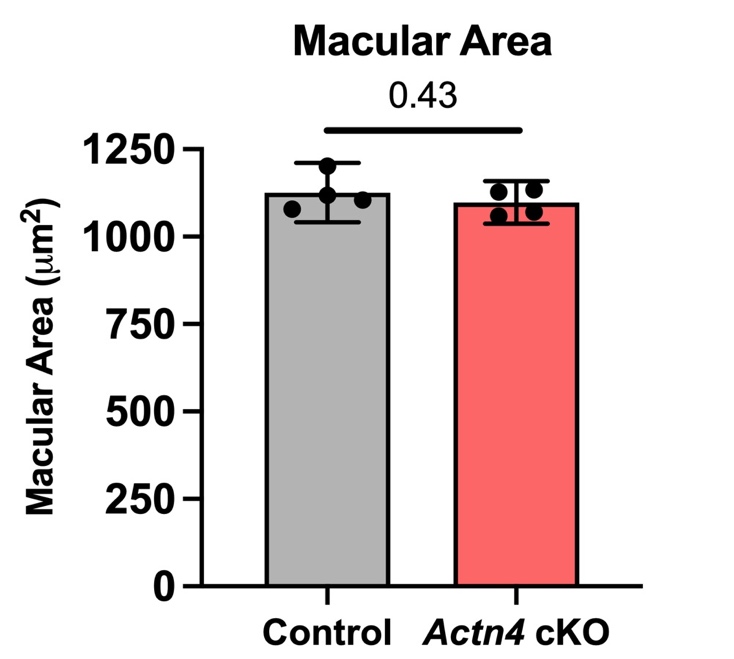


**Supplementary Figure 4.** Conditional deletion of the actin cross-linker *Actn4* in the embryonic otic vesicle does not affect area of utricles from adult mice. Each point depicts the average macular area per mouse. Graphs depict mean ± 95% confidence interval.


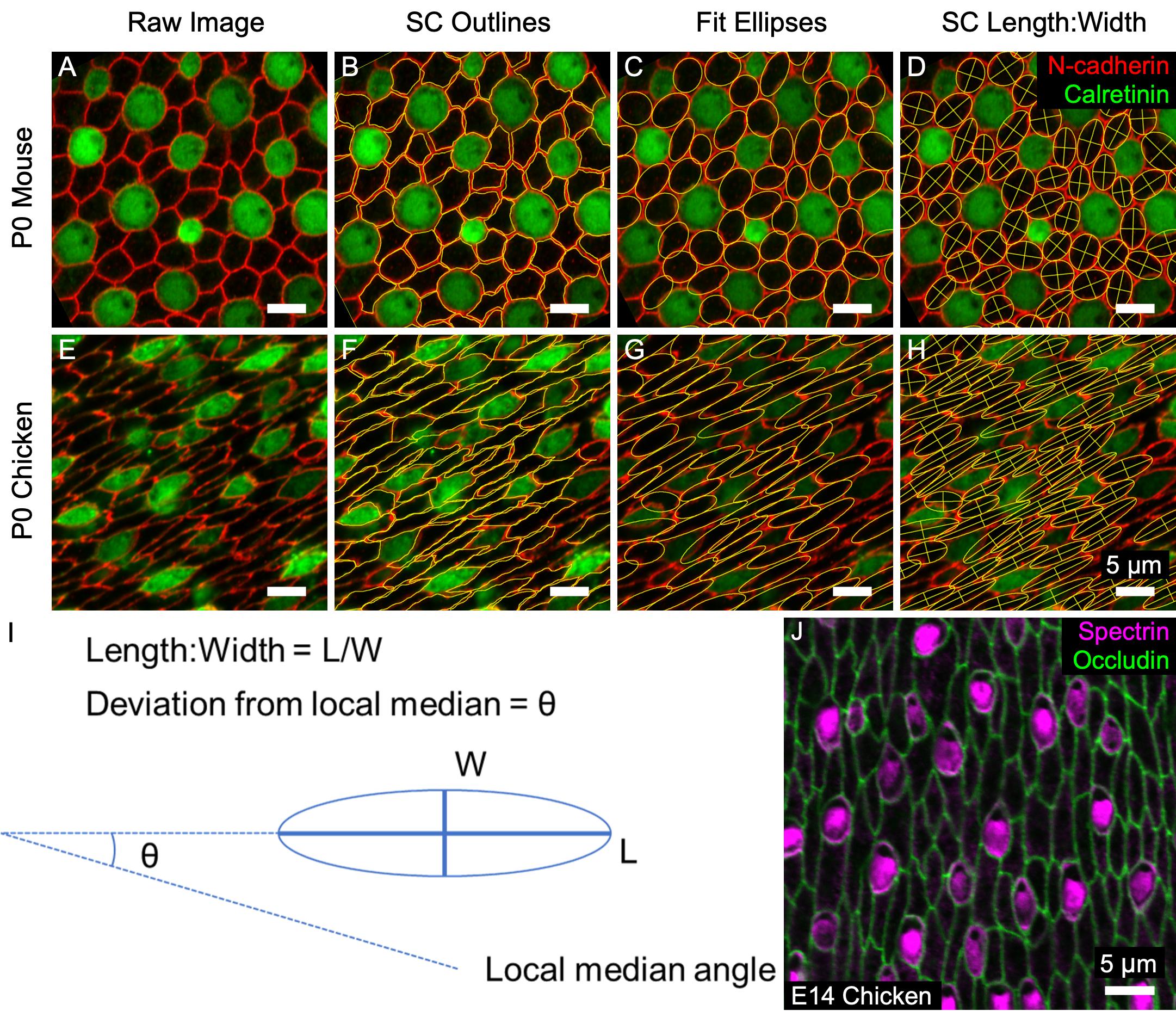


**Supplementary Figure 5.** Elongation and alignment analysis of SCs in utricles from mice and chickens. **(A, E)** Utricles were immunostained for N-cadherin to denote apical junctions and the HC marker Calretinin to distinguish HCs from SCs and imaged on a confocal microscope. **(B, F)** The apical domains of at least 30 contiguous SCs were outlined. **(C, G)** Each SC outline was fit to an ellipse. **(D, H)** The length, width, and angle of each ellipse was recorded. **(I)** The length:width ratio (L/W) provided a measure of SC elongation. To quantify local alignment, the local median angle was calculated for each image. The deviation θ of each ellipse from the local median angle was averaged to provide a measure of local alignment, ranging from 0 degrees deviation (perfect alignment) to 45 degrees deviation (random alignment). **(J)** Spectrin and occludin immunostaining of the E14 utricle show SCs are elongated and aligned in the direction of HC polarity.


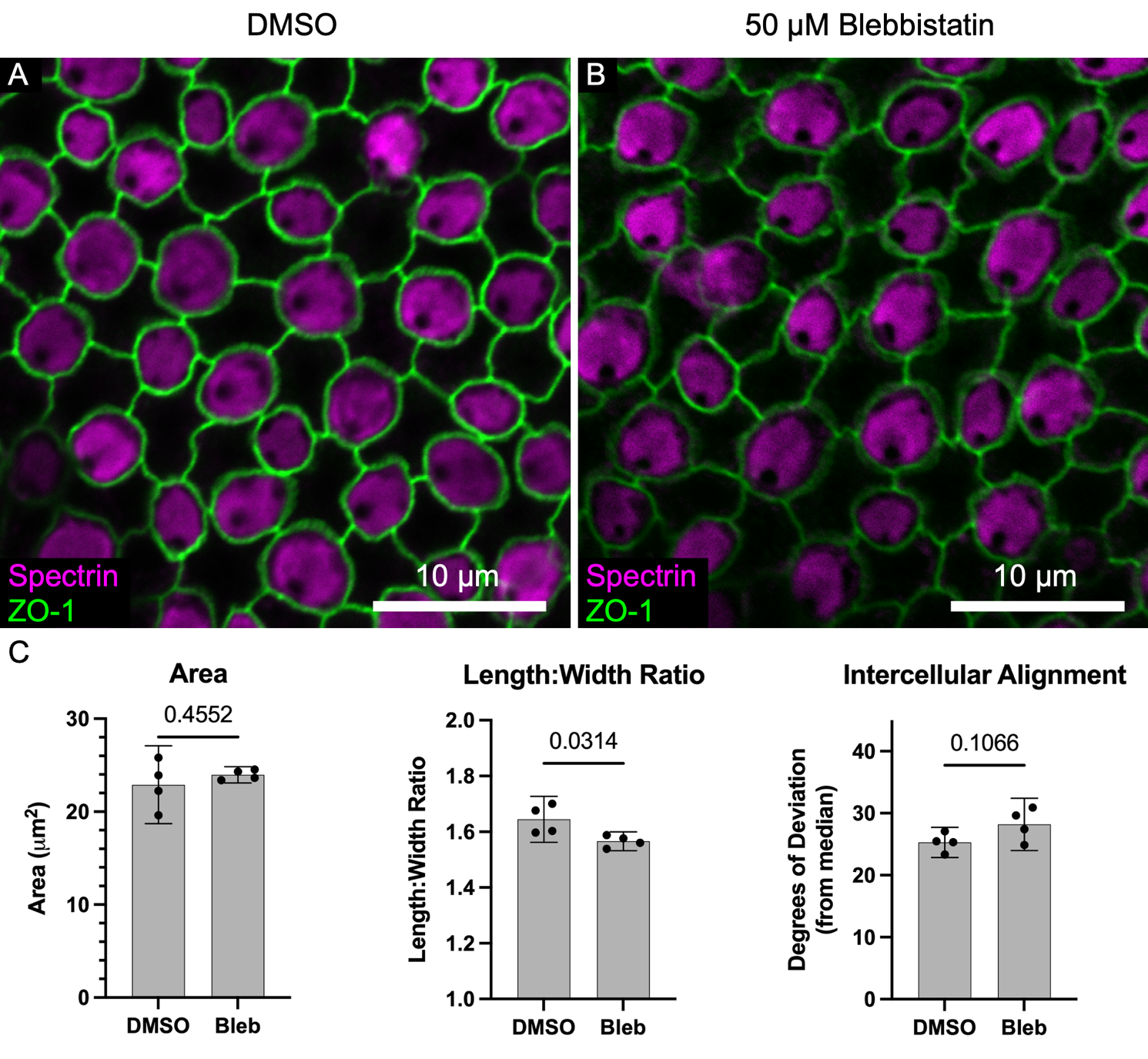


**Supplementary Figure 6.** Blebbistatin treatment of adult mouse utricles did not lead to substantial changes in SC morphology. **(A, B)** Utricles were harvested from P75 Swiss Webster mice and cultured for 5 h in the presence of 50 μM blebbistatin or DMSO vehicle control prior to fixation and immunostaining. Confocal micrographs of the epithelial surface depict spectrin labeling of HC cuticular plates and ZO-1 labeling of tight junctions. **(C)** Quantification of SC area, SC length:width ratio, and SC alignment are shown. Graphs depict mean ± 95% confidence interval, and dots denote individual utricles.
